# Deployment of Artificial Intelligence in Clinical Oral Pathology: Evidence Summary and Implementation Gaps

**DOI:** 10.1155/ancp/9281281

**Published:** 2026-08-27

**Authors:** J. H. Shazia Fathima, Mugundan Raghavelu Narendran, Mohammed Sharique Ahmed Quadri, Thilla Sekar Vinothkumar, K. A. Kamala, Mohmed Isaqali Karobari

**Affiliations:** ^1^ Global Research Cell, Dr. D. Y. Patil Dental College and Hospital, Dr. D. Y. Patil Vidyapeeth (Deemed to be University), Pimpri, Pune 411018, India, dpu.edu.in; ^2^ Department of Oral Pathology and Microbiology, Ragas Dental College and Hospital, ECR Uthandi, Chennai 600119, Tamil Nadu, India; ^3^ Department of Basic Medical Sciences, College of Medicine, Almaarefa University, Dariyah, Riyadh 13713, Saudi Arabia, um.edu.sa; ^4^ Research Center, Scientific Research and Post-Graduate Studies, Almaarefa University, Dariyah 13713, Riyadh, Saudi Arabia, um.edu.sa; ^5^ Department of Restorative Dental Sciences, College of Dentistry, Jazan University, Jazan 45142, Saudi Arabia, jazanu.edu.sa; ^6^ Department of Oral Medicine and Radiology, School of Dental Sciences, KVVDU, Satara, Karad 415110, Maharashtra, India; ^7^ Department of Conservative Dentistry and Endodontics, Saveetha Dental College and Hospital, Saveetha Institute of Medical and Technical Sciences, Saveetha University, Chennai 600077, Tamil Nadu, India, saveetha.com; ^8^ Faculty of Dentistry, University of Puthisastra, Khan Daun Penh, Phnom Penh 12211, Cambodia, puthisastra.edu.kh

**Keywords:** artificial intelligence, diagnostic accuracy, digital pathology, implementation, regulatory science, validation, whole-slide imaging

## Abstract

**Background:**

Whole‐slide imaging (WSI) has shifted pathology toward digital workflows, creating the foundation for applying artificial intelligence (AI) to diagnostic tasks. This review summarises validated AI applications in diagnostic pathology, with an emphasis on clinical performance, regulatory developments and the practical barriers that affect implementation.

**Methods:**

A structured search of PubMed, Scopus and Google Scholar identified English‐language, peer‐reviewed studies from January 2020 to May 2025. Eligible studies applied AI to diagnostic, grading or prognostic tasks in human tissue, used a pathologist‐confirmed reference standard and included external or multi‐centre validation.

**Results:**

More than 150,000 digital slides were represented across the included studies. Reported performance metrics demonstrated strong diagnostic accuracy across several validated applications. Large meta‐analyses and externally validated studies reported sensitivity values exceeding 96% and specificity above 93% for selected cancer‐detection tasks, while other studies demonstrated high agreement for Gleason grading (QWK up to 0.862) and biomarker quantification (Ki‐67 ICC 0.98).

**Conclusion:**

AI has the capacity to strengthen diagnostic pathology by improving consistency, measurement and efficiency. Moving from experimental use to routine reporting will require broad validation across centres, enhanced model transparency, strong quality‐assurance systems and close cooperation between developers and pathologists.

## 1. Introduction

Diagnostic pathology remains the gold standard reference point for cancer diagnosis and treatment planning. The field has traditionally relied on histological interpretation shaped by individual judgment and experience. Interobserver variability in grading, classification and biomarker assessment has been well documented in several pathology subspecialties, particularly in tumour grading and borderline lesions. Combined with the increasing diagnostic workload and the complexity of tissue imaging, these limitations have driven interest in digital systems that support more consistent and measurable diagnostic assessment.

Whole‐slide imaging (WSI) has driven much of the recent change in digital pathology. The conversion of conventional glass slides into high‐resolution digital images created the foundation required for computational pathology approaches to expand. Once this digital format became routine, researchers could apply artificial intelligence (AI) techniques, including deep learning and convolutional neural networks (CNNs), to routine histologic material [1]. CNNs do not rely on predefined rules; they learn visual cues from large image sets, which allows them to pick up fine morphological details that may escape even experienced observers [2–4].

Early reports showed strong performance; however, many models struggled when tested on slides produced with different scanners, staining methods, or institutional practices. This domain shift [2–4] revealed that high accuracy in controlled environments does not automatically translate into clinical reliability. For real‐world use, systems must demonstrate their performance on diverse external materials, meet regulatory expectations and integrate seamlessly into existing laboratory processes without disrupting routine work.

The years 2020–2025 saw a widespread expansion of digital pathology. WSI platforms reached clinical‐grade quality and many centres adopted digital archives for reporting, education and remote consultation. During the same period, research activity in AI for pathology accelerated, with studies focusing on cancer detection, grading and biomarker quantification [5–8]. Large datasets and pooled analyses now demonstrate that AI can achieve diagnostic performance comparable to that of expert pathologists across several tissue types [8–10].

Despite this progress, clinical adoption remains uneven. Regulatory agencies in the United States and Europe have cleared a limited number of assistive systems, including Paige Prostate and IBEX tools, which meet CE‐IVDR requirements [11, 12]. These approvals are still exceptions rather than the norm. Many models rely on limited or homogeneous datasets, which raises concerns about model interpretability and necessitates infrastructure that is not uniformly available across institutions [8, 13, 14]. This review outlines the current evidence for AI in diagnostic pathology, summarising validated use cases, diagnostic performance and regulatory developments.

## 2. Search Strategy

This review was conducted in accordance with the PRISMA‐ScR guidelines. A structured search was conducted in PubMed, Scopus and Google Scholar for materials published between January 2020 and May 2025. The search used combinations of terms related to AI (‘artificial intelligence’, ‘deep learning’, ‘machine learning’) together with terminology for digital and microscopic image analysis (‘digital pathology’, ‘whole slide image’, ‘histopathology’, ‘cytopathology’) and diagnostic applications (‘diagnosis’, ‘grading’, ‘validation’, ‘accuracy’), summarised in Table 1. Only English‐language, peer‐reviewed original studies, systematic reviews and meta‐analyses were considered.

**Table 1:** Search strategies and source information.

Database	Search strategies	Number of titles
PubMed	((‘artificial intelligence’[tiab] OR ‘deep learning’[tiab] OR ‘machine learning’[tiab] OR ‘convolutional neural network’[tiab] OR ‘CNN’[tiab]) AND (‘digital pathology’[tiab] OR ‘whole slide imaging’[tiab] OR ‘whole‐slide image’[tiab] OR ‘histopathology’[tiab] OR ‘cytopathology’[tiab]) AND (‘diagnosis’[tiab] OR ‘classification’[tiab] OR ‘grading’[tiab] OR ‘detection’[tiab] OR ‘validation’[tiab]))	3092
Scopus	Artificial intelligence, digital pathology, whole slide imaging, deep learning, diagnostic accuracy	62
Google Scholar	‘artificial intelligence’ OR ‘deep learning’ OR ‘machine learning’ ‘digital pathology’ OR ‘whole slide imaging’	5860

## 3. PCC Framework

### 3.1. Population (P)

Human histology or cytology specimens evaluated for diagnostic, grading or prognostic purposes, including whole‐slide images and other digitised preparations.

### 3.2. Concept (C)

AI approaches, such as deep learning, machine learning and CNNs, are used for tasks including detection, diagnosis, classification, grading, segmentation, or biomarker measurement in pathology.

### 3.3. Context (C)

Research performed in clinical, laboratory or retrospective digital‐archive environments that rely on digital pathology or WSI systems and report at least one quantitative diagnostic performance measure.

### 3.4. Eligibility Criteria

Studies were included when they reported the use of AI for diagnostic, grading or prognostic work in human tissue and relied on a pathologist‐confirmed reference standard. Validation was mandatory, with preference for studies that used external or multi‐centre material. Method‐only papers without clinical testing, non‐clinical experiments and conference abstracts were excluded.

### 3.5. Identification of Relevant Studies

**Figure 1:**
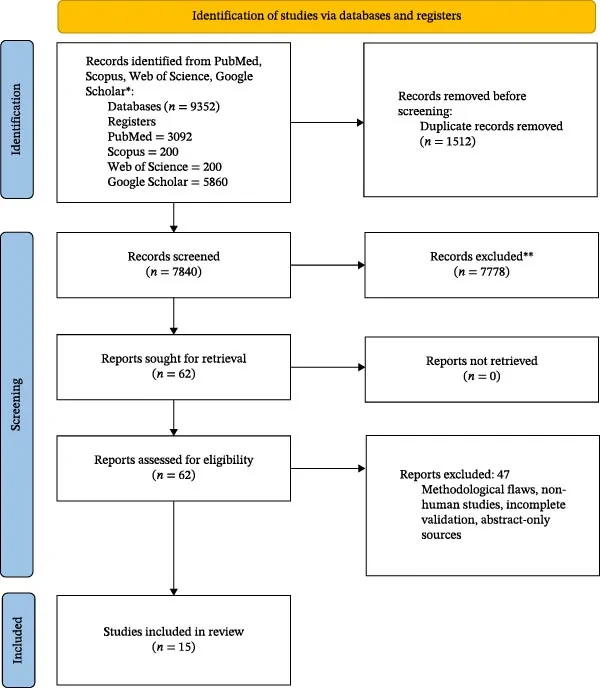
PRISMA chart.  ^∗^Consider, if feasible to do so, reporting the number of records identified from each database or register searched (rather than the total number across all databases/registers).  ^∗∗^If automation tools were used, indicate how many records were excluded by a human and how many were excluded by automation tools.

Search results from each database were screened in two stages. Titles and abstracts were assessed first to exclude items that did not involve diagnostic pathology or lacked an AI component. Full texts were reviewed for all remaining records to confirm alignment with the PCC framework and eligibility criteria. Only studies that met these requirements were taken forward for data extraction. The search and screening workflow is depicted in the PRISMA chart in Figure 1.

### 3.6. Data Charting Process

Extracted information included specimen type, the task performed by the algorithm, dataset size and diversity, the validation approach used and the performance measures reported, such as sensitivity, specificity, AUC and ICC. These variables were organised into tables to present a clear summary of the evidence. Because the included studies evaluated heterogeneous specimen types, diagnostic objectives, algorithms and validation strategies, quantitative outcomes were synthesised descriptively rather than statistically pooled across all studies. Sensitivity and specificity values discussed in this review were extracted from individual meta‐analyses or large externally validated investigations included within the evidence base and were not generated through a new aggregate meta‐analysis or formal normalisation procedure.

## 4. Results

The evolution of AI in pathology: from early morphometry to multimodal systems. Early computational work in pathology relied on rule‐driven image analysis, primarily used for basic morphometric measurements [15–17]. A significant shift occurred in the mid‐2010s when extensive collections of digitised slides became available and computational power advanced sufficiently to support deep learning. Within computational pathology research, CNN‐based models rapidly became the dominant framework for digital image interpretation because they enabled automated extraction of complex histomorphologic features from large WSI datasets.

From 2020 onward, development accelerated with broader use of WSI platforms [18], increasing interest in weakly supervised training methods and the emergence of multimodal models that link histology with genomic and clinical information [19, 20].

The evidence base identified in this review includes validated studies and meta‐analyses from groups such as McGenity et al. [8], Bulten et al. [21], Flach et al. [22], Liu et al. [23], Han et al. [24], Dy et al. [25], Choi et al. [26], Ahmed et al. [27], Dörrich et al. [28], Warin et al. [29] and several others. Combined, these reports analyse more than 150,000 digital slides across a range of tissue types, offering a broad picture of validated AI use in diagnostic pathology.

### 4.1. Mapping of Validated Diagnostic Applications

Evidence describing diagnostic performance has grown quickly in recent years. Table 2 presents a structured summary of the main findings from the included studies.

**Table 2:** Summary of included studies on validated AI applications in diagnostic pathology.

Number	Study (year)	Specimen/domain	Algorithm task	Dataset size/diversity	Validation method	Key performance metrics	Noted limitations/remarks
1	McGenity et al. (2024) [8]	Multi‐organ WSIs	Tumour detection/classification	>150,000 WSIs from 100 studies	Meta‐analysis of independent datasets	Sensitivity 96.3%; specificity 93.3%	High statistical heterogeneity; moderate–high risk of bias
2	Bulten et al. (2022) [21] (PANDA Challenge)	Prostate biopsies	Gleason grading	~ 11,000 WSIs (multi‐institutional, public)	External validation within challenge dataset	QWK 0.862 (AI vs. expert)	Generalizability and workflow integration unresolved
3	Flach et al. (2023) [22]	Breast/prostate LN sections	Lymph‐node metastasis detection	Multi‐centre internal + external validation	Head‐to‐head comparison of two CE‐marked tools	High sensitivity for macrometastases; reduced for micrometastases	Performance varies beyond intended use
4	Liu et al. (2025) [23]	Cervical cytology	Screening for CIN/carcinoma	Pooled >25,000 slides	Meta‐analysis	AUC 0.98	Workflow heterogeneity (staining, imaging)
5	Han et al. (2025) [24]	Gastric carcinoma	PD‐L1 (22C3) CPS quantification	1200 samples	Multi‐AI model integration; external validation	CCC 0.97; Sensitivity 99.2%; specificity 95.8%	Minor inter‐scanner variation
6	Dy et al. (2024) [25]	Neuroendocrine and breast cancer	Ki‐67 scoring	NR (~hundreds)	Independent comparison vs. pathologists	ICC 0.98 (AI) vs. 0.65 (pathologist)	Limited sample diversity
7	Choi et al. (2023) [26]	Breast carcinoma	TIL quantification/response prediction	700 cases	Retrospective cohort; cross‐validated	Pathologic CR 58% vs. 12% (AI TIL high vs. low)	Requires external prospective validation
8	Ahmed et al. (2025) [27]	Thyroid FNA	Benign–malignant classification	500 slides (prospective, multi‐reader)	Prospective multi‐reader study	AUC ↑ 0.79 → 0.88 (with AI)	Limited by small prospective cohort
9	Dörrich et al. (2023) [28]	HNSCC biopsies	Explainable CNN for histopath grading	NR (~hundreds)	Cross‐validation; explainability maps	Qualitative interpretability improved	Proof‐of‐concept; limited dataset size
10	Warin et al. (2022) [29]	Oral mucosal lesions	Early oral cancer detection	1000 images (single‐centre)	5‐fold CV; test split	AUC 0.82	Lacks multi‐centre validation
11	Morozov et al. (2023) [9]	Prostate biopsies	Histology identification and grading	12 studies (meta‐analysis)	Pooled > 20,000 slides	Accuracy 94%; AUC 0.96	Moderate heterogeneity
12	Wetstein et al. (2022) [10]	Breast carcinoma	Grading and survival modelling	2000 WSIs	Cross‐cohort validation	Concordant prognostic stratification	Retrospective dataset only
13	Abel et al. (2024) [15]	Pan‐cancer (nuclear morphology)	Genome instability/prognosis prediction	>5000 cases	Multi‐tumour cross‐validation	Prognostic accuracy ↑ vs. baseline	Requires clinical correlation
14	Pai et al. (2025) [30]	Mixed tumour slides	Tumour–stroma ratio estimation	1800 WSIs	Multi‐expert comparison	AI = human agreement > 0.9	Preprint; pending peer review
15	da Silva et al. (2021) [31]	Prostate cancer	Automated detection system	1200 cases (real‐world)	Prospective independent application	Clinical‐grade reproducibility reported	Early‐generation algorithm; limited tissue types

Abbreviations: AUC, area under the curve; CCC, concordance correlation coefficient; CR, complete response; CV, cross‐validation; ICC, intraclass correlation coefficient; NR, not reported; QWK, quadratic weighted kappa; TILs, tumour‐infiltrating lymphocytes; WSI, whole‐slide image.

### 4.2. Emerging Areas and Specific Domains

• Cytopathology: Strong performance has been reported for cervical cytology and thyroid FNA interpretation, although outcomes vary when staining quality and slide preparation differ between laboratories [27, 32].
• Oral Pathology: Published work remains limited because large annotated datasets are rare. Early studies have reported encouraging results for tasks such as HNSCC segmentation and the prediction of dysplasia progression (AUC 0.82) [28, 29]. A broader evaluation is limited by the low adoption of digital systems in many oral pathology centres, with estimates suggesting adoption rates below 15% [33].

### 4.3. Regulatory and Validation Landscape

Regulators have moved gradually, approving only a small number of systems after a detailed review. The FDA has cleared tools such as Paige Prostate and introduced the Predetermined Change Control Plan to guide the evaluation of adaptive algorithms [11, 34]. Despite these developments, a sizeable gap remains between laboratory‐level performance and the evidence required for routine clinical use. Prospective, multi‐centre evaluations are still limited and are essential for broader adoption.

The studies included in this review indicate a shift in computational pathology research toward validation on real diagnostic tasks. When trained on sufficiently varied and well‐annotated material, several AI models achieve performance levels comparable to expert assessment across diagnostic and grading applications [8, 21, 23]. Diagnostic accuracy estimates varied according to tissue type, algorithm design and validation methodology. Meta‐analytic evidence from cancer‐detection studies nevertheless reported sensitivity exceeding 96% and specificity above 93% in selected applications [8], indicating that some narrowly defined diagnostic tasks are approaching technical maturity.

Despite methodological heterogeneity, several consistent performance expectations emerge across the included studies. Most clinically oriented investigations considered sensitivity above 90%, specificity above 90%, AUC values approaching or exceeding 0.90 and strong concordance measures such as ICC or CCC above 0.85 as acceptable thresholds for assistive diagnostic deployment. Agreement metrics for grading applications, particularly Gleason scoring, also consistently favoured QWK values above 0.80 as indicators of clinically reliable reproducibility [21, 24]. Although these thresholds varied according to diagnostic context and clinical risk, a broad consensus was evident that AI systems intended for real‐world implementation must demonstrate stable external validation performance rather than isolated high internal accuracy.

The included studies also differed substantially in dataset scale, ranging from small single‐centre cohorts of ~500 images to meta‐analytic datasets exceeding 150,000 WSIs. This variation has important implications for model robustness and generalizability. Smaller datasets frequently reported promising internal accuracy but were more vulnerable to overfitting, spectrum bias and reduced transferability across scanners, staining protocols and institutional workflows [28, 29]. In contrast, large multi‐centre datasets and pooled analyses generally produced more stable performance estimates because they incorporated broader morphologic variability and external validation conditions [8, 21]. The evidence reviewed therefore suggests that dataset diversity and external validation may be more important for clinical reliability than model complexity alone.

### 4.4. Diagnostic and Grading Applications

Diagnostic and grading applications have progressed unevenly across organ systems, with prostate pathology emerging as the most mature and extensively validated domain. Several factors explain this trend. Prostate biopsy interpretation follows relatively standardised histomorphologic criteria through the Gleason grading system, reducing interobserver variability and enabling more reproducible annotation for algorithm training. In addition, prostate pathology generates large biopsy volumes within structured screening and diagnostic workflows, allowing the development of extensive multi‐institutional WSI repositories suitable for deep learning applications [21]. The PANDA Challenge, which included more than 11,000 WSIs from multiple centres, demonstrated that AI‐assisted Gleason grading could achieve strong reproducibility across institutions, with a QWK of 0.862 [21]. Regulatory approvals such as Paige Prostate further strengthened this field by providing one of the earliest clinically deployable validation models [11].

Breast pathology represents another rapidly developing area, particularly for lymph‐node metastasis detection, tumour grading and tumour‐infiltrating lymphocyte (TIL) quantification [22, 26]. Compared with prostate pathology, breast cancer assessment involves greater morphologic heterogeneity and more variable biomarker interpretation, which complicates model standardisation. Even so, large annotated datasets and strong clinical demand for reproducible biomarker assessment have accelerated algorithm development in this field. Studies evaluating TIL quantification and response prediction have reported clinically relevant prognostic stratification, although prospective external validation remains limited [26].

Cytopathology applications, including cervical cytology and thyroid FNA interpretation, have shown encouraging diagnostic performance [23, 27]. However, these systems remain highly sensitive to differences in staining quality, fixation methods, smear preparation and image acquisition protocols between laboratories. Unlike histologic WSIs, cytology specimens often contain overlapping cells, variable background artefacts and inconsistent focal planes, creating additional computational challenges that reduce transferability across institutions [32].

Progress in oral and head‐and‐neck pathology remains comparatively limited. Although early studies demonstrated promising performance for dysplasia prediction and HNSCC segmentation [28, 29], broader validation is constrained by limited access to large annotated datasets and lower adoption of digital pathology infrastructure in oral pathology centres [33]. Histologic heterogeneity, relatively low case volumes for specific lesions and inconsistent grading criteria across studies further complicate model development. Similar barriers affect several smaller pathology subspecialties where dataset scarcity and limited multi‐centre collaboration restrict robust external validation.

Across organ systems, the transition from experimental algorithms to clinically reliable tools depends on overcoming several shared barriers. These include the need for larger multi‐centre datasets, harmonisation of staining and scanning protocols, transparent validation standards, improved interpretability and prospective workflow‐based studies performed under real clinical conditions. The evidence reviewed suggests that successful clinical translation is more likely in disease settings with standardised diagnostic criteria, high specimen volume and established digital infrastructure.

### 4.5. Translational Barriers

Although the diagnostic performance reported for several AI systems is encouraging, routine implementation remains substantially behind research development. Histopathology diagnosis in most institutions continues to rely on direct microscopic examination by pathologists, and AI deployment is largely dependent on prior adoption of digital pathology workflows. Recent analyses of digital pathology implementation have emphasised that the principal challenge is not algorithm performance alone but the limited penetration of WSI into routine clinical practice. Factors affecting adoption extend beyond technical validation and include organisational culture, workforce readiness, workflow redesign, regulatory requirements, infrastructure investment and long‐term maintenance costs. Studies evaluating real‐world implementation have consistently shown that successful adoption requires extensive change‐management processes, training programs, pathologist engagement and institutional commitment before AI tools can be integrated into routine reporting. Consequently, many AI systems remain confined to pilot projects, validation studies, or narrowly defined assistive applications rather than widespread clinical deployment [35–40].

**Figure 2:**
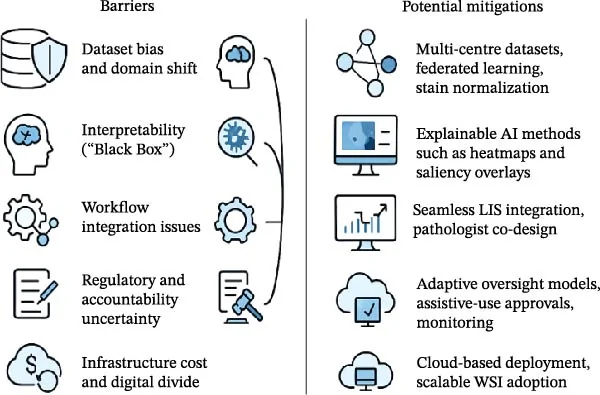
Key barriers to implementing AI in diagnostic pathology and corresponding mitigation strategies.

Progress remains uneven, even in areas where accuracy metrics look strong. The significant challenges identified across the included studies, along with corresponding mitigation strategies, are summarised in Table 3 and Figure 2. One recurring problem is the heavy dependence on single‐centre cohorts, which narrows the range of training examples and weakens the performance when models are tested elsewhere [8]. Groups have begun experimenting with approaches such as federated learning and structured data augmentation to broaden the range of training materials while maintaining patient information protection. These strategies still need coordinated involvement from several institutions and transparent agreements on how data are handled.

**Table 3:** Key translational barriers and potential mitigating strategies.

Challenge	Evidence of the problem	Potential mitigating strategies
Dataset bias and lack of generalizability	Meta‐analyses report high risk of bias due to single‐centre, homogenous datasets [8].	Multi‐centre consortia; federated learning; synthetic data augmentation.
The ‘Black Box’ problem and lack of interpretability	Pathologist surveys consistently cite interpretability as a major barrier to trust and adoption.	Explainable AI (XAI) techniques; ‘Human‐in‐the‐loop’ workflows; algorithmic literacy training.
Workflow integration and usability	Successful adoption requires seamless integration into LIS without disrupting diagnostic efficiency [41].	Co‐design with pathologists; standardised interoperability protocols (e.g., DICOM); prospective workflow studies.
Resource and equity constraints	High cost of digital infrastructure creates a ‘digital divide’,limiting access in low‐resource settings [42].	Cost‐effective scanning solutions; validation on globally diverse datasets; public‐private partnerships.

Concerns regarding model interpretability continue to influence the clinical adoption of AI systems in pathology. Multiple reviews and implementation studies have identified limited transparency, uncertainty regarding error attribution and difficulty correlating algorithmic outputs with conventional morphologic reasoning as recurring barriers to pathologist trust and acceptance [8, 13, 14]. Explainable‐AI approaches, including attention maps, saliency overlays and class‐activation visualisations, have therefore been developed to improve interpretability by linking predictions to identifiable histologic regions [13, 28]. Current evidence from workflow‐based implementation studies suggests that assistive or ‘human‐in‐the‐loop’ models, where pathologists retain responsibility for final interpretation while reviewing algorithm‐generated outputs, remain the most practical near‐term strategy for clinical deployment rather than fully autonomous reporting systems [8, 11, 31].

Practical deployment also depends heavily on interoperability with existing laboratory information systems (LIS), digital slide archives and reporting workflows [43]. Several implementation studies have shown that inefficient integration increases reporting delays, workflow fragmentation and user resistance, particularly when AI outputs require manual transfer between platforms [14, 33]. Adoption is therefore more successful when AI systems are embedded directly within routine digital pathology environments using standardised interoperability frameworks, such as DICOM for pathology imaging and structured reporting protocols. Despite growing commercial interest, formal prospective workflow studies evaluating turnaround time, usability, diagnostic concordance and cost‐effectiveness remain relatively limited.

Access to digital pathology infrastructure remains highly uneven across institutions and geographic regions. The barriers to implementation extend beyond infrastructure. Surveys and implementation studies have identified cultural resistance to workflow change, concerns regarding diagnostic responsibility, uncertainty about regulatory oversight and limited confidence in algorithmic decision‐making as important obstacles to adoption. Pathologists frequently express greater willingness to use AI as a decision‐support tool rather than as an autonomous diagnostic system. In addition, laboratory administrators must justify substantial investments in slide scanners, image storage systems, network capacity, software licencing, validation procedures and ongoing technical support. The economic return on these investments may be difficult to demonstrate immediately, particularly in smaller pathology services and resource‐constrained healthcare systems. These factors help explain why successful research studies have not yet translated into universal clinical implementation [35–40]. Multiple studies have identified the cost of slide scanners, high‐capacity data storage, network bandwidth requirements and long‐term maintenance as major barriers to implementation, particularly in smaller laboratories and resource‐constrained healthcare systems [14, 33]. These infrastructural limitations directly affect the availability of large annotated datasets, the feasibility of WSI adoption and the ability to perform external validation across diverse populations. Broader implementation is therefore more likely when supported through collaborative multi‐institutional networks, public–private funding initiatives, scalable cloud‐based storage solutions and validation studies that include centres from varied resource settings. Without these measures, digital pathology expansion may inadvertently widen existing disparities in diagnostic access and technological capacity.

### 4.6. Regulatory and Validation Landscape

Regulatory progress continues but remains measured. The FDA’s authorisation of Paige Prostate and the release of the Predetermined Change Control Plan for adaptive AI systems [11, 34] indicate growing clarity regarding the oversight of these tools. The CE‐IVDR authorisation of the IBEX Prostate platform [12] exhibits a similar trend in Europe. Nevertheless, most published algorithms have not yet undergone regulatory review. The persistent gap between technical success and clinical qualification highlights the need for multi‐centre prospective studies and transparent reporting of real‐world outcomes.

## 5. Future Directions

Recent work is shifting toward multimodal and generative approaches that merge histologic images with genomic and clinical information to build more integrated disease models [19, 20].

Emerging evidence further suggests that the next phase of computational pathology will rely on multimodal AI systems that integrate histomorphologic features with immunohistochemical (IHC) biomarkers and molecular or genomic data (Table 4). Rather than analysing tissue morphology alone, these models combine complementary sources of biological information to improve diagnostic accuracy, tumour classification, prognostic stratification and prediction of therapeutic response. Several studies have demonstrated that integrating digital histopathology with molecular profiling enables AI models to identify clinically relevant tumour subtypes, infer genomic alterations directly from tissue morphology and support precision oncology by linking morphologic patterns with actionable biomarkers. Likewise, combining AI‐assisted quantitative assessment of IHC markers such as Ki‐67, PD‐L1, ER, PR and HER2 with histologic image analysis has the potential to reduce interobserver variability while providing more reproducible biomarker evaluation for treatment selection. As multimodal datasets become increasingly available through large collaborative initiatives, the integration of histologic, immunophenotypic and molecular information is expected to become a cornerstone of future computational pathology and personalised cancer diagnostics.

**Table 4:** Overview of AI applications in biomarker quantification and prognostication.

Specimen type/domain	Platform/algorithm	Primary application	Reported clinical utility/performance
Gastric carcinoma	Multi‐AI PD‐L1 CPS integration model (Han et al. [24])	Quantitative PD‐L1 (22C3) CPS scoring	CCC 0.97; sensitivity 99.2%; specificity 95.8% for CPS ≥1 [24]
Breast and neuroendocrine tumours	Ki‐67 AI quantification systems (Dy et al. [25])	Automated Ki‐67 scoring	AI ICC 0.98 versus pathologist ICC 0.65
Breast carcinoma	Deep‐learning stromal TIL analysis (Choi et al. [26])	TIL quantification and response prediction	Pathological complete response 58% vs. 12% between highest and lowest AI‐defined TIL quartiles [26]
Thyroid cytology	WSI‐assisted thyroid FNA AI model (Ahmed et al. [27])	Benign–malignant classification	AI‐assisted pathologist AUC improved from 0.79 to 0.88 [27]
Breast carcinoma	Roche VENTANA uPath Ki‐67 (MIB‐1)	Automated Ki‐67 proliferation assessment	Reduced interobserver variability and improved scoring standardisation in breast cancer
NSCLC/multiple solid tumours	Roche VENTANA PD‐L1 (SP263) algorithm	PD‐L1 expression quantification	Improved reproducibility and standardised immune checkpoint biomarker interpretation
Breast pathology	Indica Labs HALO Breast IHC	ER, PR, HER2 and Ki‐67 quantification	High concordance with manual pathologist scoring in digital breast pathology workflows
Lung carcinoma	Indica Labs HALO PD‐L1	Automated PD‐L1 scoring	Enhanced reproducibility for tumour proportion scoring and immune‐cell analysis
Prostate pathology	Ibex Medical Analytics platform	Prostate cancer detection and grading	CE‐IVDR approved workflow integration with assistive diagnostic support
Multi‐organ digital pathology	Lunit SCOPE	Detection, segmentation and biomarker analysis	Multi‐cancer AI platform with validated deployment in digital pathology environments
Breast, lung and gastrointestinal pathology	Aiforia AI models	Biomarker quantification and tissue classification	Cloud‐based deep learning workflow with multi‐tissue application support

By linking these data types, researchers aim to predict outcomes, treatment responses and other clinically useful endpoints, thereby moving the focus beyond image‐based pattern recognition. Federated learning initiatives and large shared datasets are likely to play a key role in addressing the privacy constraints and reducing the variability introduced by institution‐specific practices. As these technologies advance, pathologists will need to be familiar with algorithm behaviour and limitations, supporting a working model in which human expertise and computational tools function together rather than in opposition. In routine diagnostics, the practical value of AI will rest on its ability to strengthen rather than displace professional judgment. Gains in precision, consistency and reporting efficiency are expected only when systems operate within an integrated digital environment where pathologists remain responsible for final interpretation.

### 5.1. Limitations of the Review

This review provides an overview of validated AI applications in pathology, but several important limitations must be acknowledged. First, although the included studies represent influential and highly cited work in computational pathology, the final evidence base remained relatively small and heterogeneous, with ~15 major studies forming the core comparative dataset. This limits the ability to draw universally generalisable conclusions across all pathology subspecialties, diagnostic settings and AI architectures.

A further limitation is the broader publication bias that currently affects digital pathology research. Many commercially developed algorithms, proprietary validation datasets and industry‐led clinical evaluations remain unpublished or only partially accessible in the peer‐reviewed literature. As a result, publicly available studies may overrepresent high‐performing systems while underreporting failed implementations, negative findings or workflow‐related limitations. This selective visibility complicates the independent assessment of real‐world reproducibility and external validity.

Several included studies also relied heavily on single‐centre retrospective cohorts or centrally curated repositories such as The Cancer Genome Atlas (TCGA). Although these resources provide large annotated datasets suitable for algorithm development, they may not fully represent the variability encountered in routine clinical practice. TCGA‐derived images are often generated under controlled scanning and staining conditions, whereas real‐world pathology workflows involve substantial inter‐institutional variation in tissue processing, fixation, scanner calibration and reporting practices. Models trained predominantly on centralised or homogeneous datasets, therefore, remain vulnerable to domain shift and reduced performance when applied across different laboratories and patient populations [8, 31].

The included literature also varied substantially in methodology, dataset quality, validation strategy and reporting standards [8, 9]. Many algorithms lacked prospective multi‐centre validation under routine clinical conditions, and several studies depended primarily on retrospective internal testing [21, 31]. Restricting the search to English‐language publications from 2020 to 2025 may additionally have excluded relevant earlier or non‐English evidence. Digital infrastructure limitations in subspecialties such as oral pathology and cytopathology further reduce the availability of large public datasets and robust external validation studies [27–29, 32, 33]. Collectively, these factors limit generalizability but still provide a representative overview of the current evidence base, translational barriers and implementation challenges surrounding AI in diagnostic pathology.

## 6. Conclusion

AI in diagnostic pathology has progressed beyond experimental development and now demonstrates clinically meaningful performance in selected applications, particularly in cancer detection, grading and biomarker quantification. The strongest evidence currently exists in pathology domains with standardised grading systems, high specimen volumes and large multi‐centre datasets, such as prostate and breast pathology. Several assistive AI systems have already entered regulated clinical workflows, although implementation remains uneven across subspecialties and institutions. Nevertheless, widespread adoption remains constrained by the pace of digital pathology implementation, workforce acceptance, institutional investment requirements and the need for sustainable integration into existing laboratory workflows [35–40]. The findings of this review indicate that AI is presently most effective as a decision‐support tool integrated within pathologist‐supervised digital workflows rather than as a replacement for expert interpretation. Broader clinical adoption will depend on prospective multi‐centre validation, improved interoperability with laboratory systems, greater access to digital infrastructure and the use of diverse real‐world datasets that strengthen external generalizability and reduce implementation bias.

## Author Contributions

Conceptualisation, methodology, data curation, formal analysis, writing – original draft: J. H. Shazia Fathima, Mugundan Raghavelu Narendran, Mohammed Sharique Ahmed Quadri, Thilla Sekar Vinothkumar and K. A. Kamala. Conceptualisation, methodology, supervision, visualisation, writing – review and editing: Mohmed Isaqali Karobari.

## Funding

No funding was received for this manuscript.

## Ethics Statement

The authors have nothing to report.

## Consent

The authors have nothing to report.

## Conflicts of Interest

The authors declare no conflicts of interest.

## Data Availability

Data sharing is not applicable to this article, as no datasets were generated or analysed during the current study.
